# Ophthalmic Manifestations of Syphilis

**Published:** 1909-04-17

**Authors:** 


### Ophthalmology.

### OPHTHALMIC MANIFESTATIONS OF SYPHILIS.

Iritis, or irido-cyclitis, is the most frequent syphilitic lesion within the eye during the acute stage; choroditis, or retino-choroiditis the most frequent chronic inflammation from the same cause. The large majority of cases of iritis met with are due to syphilis, that is, of primary iritis, not due to trauma. It is an early secondary manifestation—one of the earliest—and is invariably accompanied by cyclitis. The eye becomes red and uncomfortable, with ciliary injection, hazy anterior chamber due to exudation in the aqueous; there is a muddy appearance of the iris, which loses its brilliancy and becomes very sluggish in its reactions. Later a yellowish nodule may develop in the iris tissue in one of two situations, either at the attached margin in the angle of the anterior chamber, or at the free pupillary margin. Nodules which appear within the width of the iris are rarely syphilitic. The iritis which very often appears during the tertiary stage of the disease is not usually accompanied by these nodules, but at times nodules develop which must be looked upon as gummatous. Syphilitic iritis also occurs in intra-uterine life, and a child is occasionally born with posterior synechiæ and a bound-down pupil. Nodules in the iris are due either to syphilis, in which case they disappear rapidly under a course of mercurial inunction, to tubercle, or to sarcoma. In clearing up they may leave no scar, or that portion of the iris occupied by them may become atrophic and very thin.

Iritis occurs in hereditary syphilis, almost invariably accompanied by interstitial keratitis. It is extremely rare to come across a case of interstitial keratitis without iritis accompanying it, though the latter is sometimes impossible to diagnose owing to the haze of the cornea. This form of iritis occurs in children from three to thirteen years of age, the ordinary specific iritis in adults, and the form occurring as a tertiary manifestation from thirty-five upwards. If a patient above forty develops specific iritis he will probably die of some syphilitic trouble.

Cyclitis occurs at times as a secondary manifestation, with but little if any affection of the iris, and patients then seek advice on account of greatly diminished vision. In such cases, frequently the only signs to be found are marked floating opacities in the vitreous, and keratitis punctata (K.P. so called) on the back of the cornea, with some tenderness on pressure over the ciliary body, and neuralgic pain, usually at night, round the orbit. This condition may continue for many months, and in the end, if not relieved, may give rise not only to total loss of vision but also to shrinking of the eyeball. Even if such a disastrous result does not happen, the lens may suffer in its nutrition, and quite early become cataractous, or the iris may become involved, get bound down, and glaucoma supervene, or the pupil become blocked by lymph. Very fine dust-like opacities in the vitreous—so fine as to be seen only with the direct method of using the ophthalmoscope and a plane mirror in place of the ordinary small concave mirror, together with a moderately high convex lens—are diagnostic of syphilis, or of there having been specific cyclitis at some previous time. The vitreous cannot become inflamed from syphilis, but is chiefly affected by exudation into it from inflammations of the ciliary body and retina.

Inflammation of the retina occurs as a direct result of syphilis, sometimes congenital, but most usually acquired. Syphilitic retinitis is found in two forms, and as a rule is accompanied by inflammation of the choroid. The retina may lose its transparency and become hazy throughout its entire extent; the haziness is most marked in the neighbourhood of the optic nerve, since the retinal layers are thicker here than elsewhere; or circumscribed patches of haziness may appear which eventually become definite patches of exudation projecting into the vitreous, isolated from one another, and appearing in different parts of the retina, sometimes attacking the region of the macula only. The vitreous quite early becomes filled with opacities, rendering ophthalmoscopic examination difficult; but the exudations, if of large size, can usually be seen on account of the difference in colour, in spite of the vitreous haze. The more general diffuse form may continue for a very long time without any apparent alteration, but finally changes in the retinal pigment take place, and the whole retina becomes occupied by fine dotted pigment, which runs together until the appearance is similar to that known as retinitis pigmentosa. The circumscribed form finally settles into a whitish mass of connective tissue, with but little pigment round it, or the choroid having become involved, shows the distinctive features of choroiditis—a white punched-out centre surrounded by masses of dark brown or black pigment, the centre white part indicating where tne choroid has atrophied and the glistening sclerotic shows through. Choroiditis is one of the most frequently observed intra-ocular inflammations caused by syphilis, and it is found also as a congenital defect in the subjects of the hereditary affection. Isolated patches are at times met with, but it is more usual for succeeding foci of inflammation to follow the first at varying intervals of time, until the usual clinical picture of disseminated choroiditis presents itself. The patches appear in the periphery first, and succeeding attacks bring others nearer to the central region, and sometimes directly to the macula, at once destroying central vision; though this order may not always be adhered to, it is not uncommon, and in other cases numbers of peripherally placed patches have been found in or near the macula, the result of previous inflammatory attacks. After interstitial keratitis with irido-cyclitis (when the cornea has cleared sufficiently to allow of ophthalmoscopic examination) patches of disseminated choroiditis are found dotted over the fundus.

The optic nerve is affected directly by syphilis, as in the primary optic atrophy of tabes; it also shares in retinal inflammation, and may atrophy after a severe attack of retinitis; neuritis or choked disc (so called) may result from intracranial gumma.

